# A Case of Spontaneous Tumor Lysis Syndrome With Acute Kidney Injury and the Timing of Chemotherapy

**DOI:** 10.7759/cureus.85342

**Published:** 2025-06-04

**Authors:** Xiaowei Malone, Bakr Alhayek, Orlando Telleria, Juan F Garza

**Affiliations:** 1 Internal Medicine, AdventHealth Tampa, Tampa, USA; 2 Oncology, Florida Cancer Specialists, Tampa, USA

**Keywords:** acute kidney injury, chemotherapy, high-grade lymphoma, hiv-associated lymphoma, spontaneous tumor lysis syndrome

## Abstract

Tumor lysis syndrome (TLS) is an oncological emergency that can lead to acute kidney injury (AKI), cardiac arrhythmias, seizures, and death. TLS typically arises after initiation of chemotherapy. However, spontaneous TLS (STLS) can occur rarely. Here, we present the case of a 56-year-old male with HIV-associated stage IV diffuse large B-cell lymphoma, who presents with STLS with severe AKI. With hemodialysis, febuxostat, and dose-adjusted chemotherapy, successful resolution of metabolic derangements and return of his baseline kidney function was achieved. Our case aims to provide insight into when to initiate chemotherapy in STLS patients with severe AKI.

## Introduction

Tumor lysis syndrome (TLS) is an oncological emergency that arises from the sudden release of intracellular ions and nucleic acids into the circulation following the rapid destruction of tumor cells, typically in the context of malignancies with high proliferative indices (i.e., a high percentage of cells actively dividing, often estimated by Ki-67 staining or mitotic counts) [1]. It is classically defined by hyperkalemia, hyperphosphatemia, hyperuricemia, and hypocalcemia, each of which can lead to life-threatening sequelae if not promptly identified and treated [1,2]. Although TLS most frequently develops within 12 to 72 hours after the initiation of chemotherapy in hematologic cancers, a subset of patients may experience spontaneous TLS (STLS) in the absence of active oncologic treatment [3]. The high levels of circulating uric acid and phosphate in TLS are particularly concerning, given their propensity to form precipitates within renal tubules, thereby contributing to obstructive nephropathy and acute kidney injury (AKI) [4]. Epidemiologically, AKI complicates up to 64% of TLS admissions, with mortality rising from roughly 20% overall to nearly 40% when dialysis is required [5]. Despite the severity of STLS, there remains a lack of clear guidelines to direct the management of patients who present with significant metabolic derangements and tumor burden without prior chemotherapy exposure [6]. Although initiating cytotoxic therapy can help decrease tumor volume and mitigate further metabolic release, it may also exacerbate preexisting electrolyte imbalances and potentially worsen renal dysfunction through additional tumor cell lysis [7]. Furthermore, certain chemotherapeutic agents themselves exhibit nephrotoxic properties, creating an additional layer of complexity in choosing the optimal timing and regimen for patients with STLS [8].

## Case presentation

**Figure 1:**
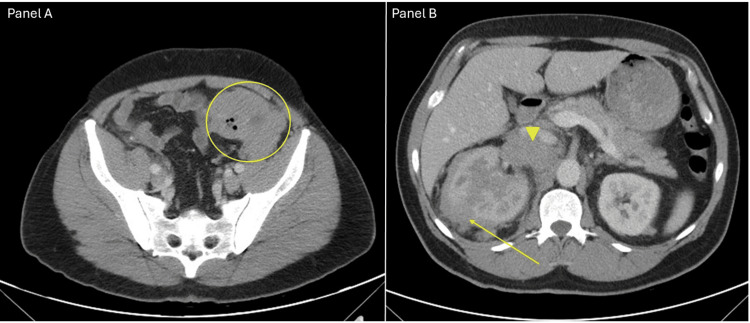
Axial contrast-enhanced CT images showing the primary sigmoid colon mass and its metastatic spread Panel A (pelvic level) demonstrates a bulky exophytic mass arising from the sigmoid colon in the left lower quadrant, measuring approximately 6.4 × 6.5 × 10.9 cm (yellow circle). Panel B (upper abdominal level) reveals metastatic disease, including a heterogeneously enhancing right adrenal lesion measuring 1.7 × 3.4 cm (solid yellow arrow) and extensive retroperitoneal/right retrocrural lymphadenopathy, the largest nodal conglomerate medial to the upper pole of the right kidney measuring 5.7 × 4.2 cm (yellow arrowhead).

**Figure 2:**
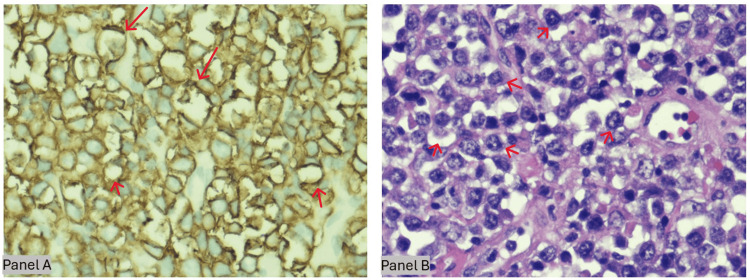
Histopathologic and immunophenotypic features of the sigmoid colon mass (original magnification ×60) Panels A-B (×60). High-power histology of the sigmoid-colon mass. A) H&E shows sheets of large atypical lymphoid cells with vesicular chromatin, prominent nucleoli, brisk mitoses, and scattered tingible-body macrophages (“starry-sky” red arrows), infiltrating through muscularis into pericolic fat - features of diffuse large B-cell lymphoma. B) CD20 immunostain reveals diffuse strong membranous positivity (red arrows), confirming mature B-cell lineage.

**Figure 3:**
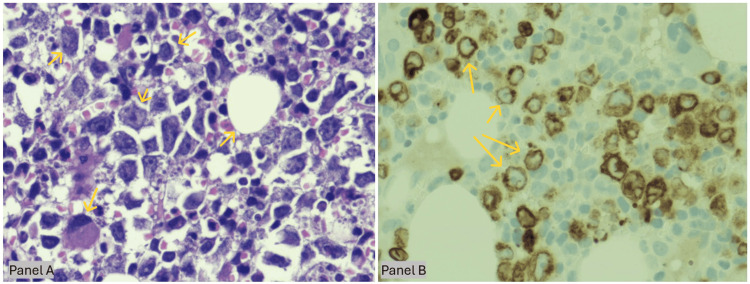
Bone marrow involvement by diffuse large B-cell lymphoma (original magnification ×60) Panels A-B, bone marrow core biopsy showing diffuse large B-cell lymphoma. A) H&E reveals near-total replacement of marrow by large pleomorphic lymphoid cells (yellow arrows) with vesicular chromatin, prominent nucleoli, and scattered tingible-body macrophages, creating a patchy starry-sky pattern. B) Corresponding CD79a immunostain demonstrates strong granular membranous/cytoplasmic positivity in the neoplastic cells (yellow arrows), confirming mature B-cell lineage, while native hematopoietic elements remain negative.

A 56-year-old male (BMI 24.6) with hypertension and well-treated HIV infection on Biktarvy with a near undetectable viral load of 22.5 copies/mL (reference <20) but severe immunosuppression reflected by an absolute CD4 count of 79 cells/µL who initially presented with progressive abdominal pain and altered bowel habits for one week. Diagnostic imaging and endoscopic evaluation revealed a mass in the sigmoid colon, and surgical pathology later confirmed a diagnosis of diffuse large B-cell lymphoma (DLBCL). Further staging investigations, including imaging and bone marrow biopsy, demonstrated stage IV disease involving the sigmoid colon, bone marrow, and retroperitoneal lymph nodes (Figures 1-3). He subsequently underwent sigmoid mass resection and ureteral stent placement to address extrinsic compression of the ureter.

Ten days postoperatively, the patient returned to the emergency department with severe generalized weakness, profound fatigue, and reduced oral intake. On initial evaluation, he was found to be hypotensive with a blood pressure of 92/60 mmHg and tachycardic with a heart rate of 116 beats per minute. Laboratory investigations (Table 1) revealed markedly elevated blood urea nitrogen (127 mg/dL) and serum creatinine (9.8 mg/dL), indicative of AKI. Serum potassium measured 6.1 mmol/L, serum phosphate was 7.9 mg/dL, and uric acid was 16.7 mg/dL. Additional lab abnormalities included a lactic acid level of 11.9 mmol/L and lactate dehydrogenase exceeding 2,500 U/L, suggesting extensive tumor turnover and cellular breakdown.

**Table 1:** Laboratory findings of the patient on admission consistent with spontaneous tumor lysis syndrome

Lab parameter	Patient value	Reference range
Blood urea nitrogen (BUN)	127 mg/dL	6-20 mg/dL
Creatinine	9.8 mg/dL	0.6-1.3 mg/dL
Potassium	6.1 mmol/L	3.5-5.1 mmol/L
Phosphorus	7.9 mg/dL	2.5-4.5 mg/dL
Uric acid	16.7 mg/dL	3.4-7.0 mg/dL
Lactate	11.9 mmol/L	0.5-2.2 mmol/L
Lactate dehydrogenase (LDH)	>2,500 UI/L	125-250 UI/L
Calcium	6.8	8.8-10.5 mg/dL
Corrected calcium to albumin	7.8	8.8-10.5 mg/dL

These metabolic disturbances were consistent with stage 3 TLS per the Cairo-Bishop classification [9]. Notably, the absence of recent chemotherapy administration led to a presumptive diagnosis of STLS, a rare phenomenon that can occur in highly proliferative lymphomas. The patient was admitted to the intensive care unit for continuous hemodynamic and electrolyte monitoring. Given the severity of his AKI and life-threatening electrolyte derangements, urgent hemodialysis was initiated to control hyperkalemia, hyperphosphatemia, and hyperuricemia.

Although rasburicase is the preferred agent to rapidly reduce uric acid levels, it was unavailable. The team therefore administered febuxostat (40 mg daily) to inhibit xanthine oxidase activity and reduce further uric acid production. High-dose corticosteroids were also introduced to diminish tumor-related inflammation. On hospital day 3 and despite initial concerns that chemotherapy might exacerbate AKI by promoting additional tumor cell lysis, the oncology service proceeded with a dose-adjusted R-EPOCH (rituximab, etoposide, prednisone, vincristine, cyclophosphamide, doxorubicin) protocol delivered over five consecutive days: rituximab on day 1; a 96-hour continuous infusion of etoposide, doxorubicin, and vincristine (days 1-4); cyclophosphamide on day 5; and oral prednisone on days 1-5. Twenty-four hours after completing the systemic cycle, the patient received a single dose of intrathecal methotrexate for CNS prophylaxis.

During the initial course of chemotherapy, the patient remained under close observation in the intensive care unit. Electrolyte correction and ongoing renal replacement therapy supported his rapid clinical improvement. Over the following week, his serum creatinine steadily declined, and electrolyte abnormalities normalized (Table 2). By the time of discharge, his renal function had returned to near-baseline levels, and dialysis was discontinued, and he was discharged with plans for outpatient cycles every 21 days. He completed cycles 2-4 uneventfully; interim PET-CT after cycle 3 showed a partial metabolic response. Five months later, however, he developed leptomeningeal relapse with obstructive hydrocephalus requiring ventriculoperitoneal shunting, Ommaya reservoir placement, and transfer to an academic center for weekly intrathecal therapy and high-dose methotrexate. Further clinical details after transfer are unavailable because our institution does not have access to the receiving center's records.

**Table 2:** Laboratory findings of the patient on discharge

Lab parameter	Patient value	Reference range
Sodium	134 mmol/L	133-143 mmol/L
Potassium	3.8 mmol/L	3.6-5.2 mmol/L
Chloride	98 mmol/L	98-112 mmol/L
Blood urea nitrogen (BUN)	16 mg/dL	7-18 mg/dL
Creatinine	1 mg/dL	0.6-1.3 mg/dL
Phosphorus	2.2 mg/dL	2.5-4.5 mg/dL
Calcium	8.4 mg/dL	8.8-10.5 mg/dL
Uric acid	3.7 mg/dL	3.4-7.0 mg/dL
Lactate	1.1 mmol/L	0.5-2.2 mmol/L
Lactate dehydrogenase (LDH)	750 UI/L	125-250 UI/L

## Discussion

STLS is a rare but critical complication of highly proliferative malignancies, particularly hematologic neoplasms, characterized by the abrupt release of intracellular contents into the bloodstream in the absence of chemotherapy [10,11]. This syndrome involves the liberation of potassium, phosphate, and nucleic acids, which are metabolized to uric acid, culminating in a constellation of life-threatening abnormalities that include hyperkalemia, hyperphosphatemia, hypocalcemia, and AKI [10,11]. Although TLS most often manifests after the initiation of cytotoxic therapy, STLS can develop spontaneously in patients with high tumor burdens or rapidly dividing cell populations; thus, it may present with severe metabolic derangements before any prophylactic measures, such as hydration or urate-lowering therapy, can be implemented [10,12].

The absence of prophylaxis in STLS often results in higher levels of uric acid and phosphate compared to chemotherapy-induced TLS, thereby escalating the risk of renal tubule obstruction from uric acid and calcium phosphate precipitates [10,13]. When AKI ensues, clinicians face the dilemma of whether to initiate, delay, or dose-adjust chemotherapy, given that additional tumor cell lysis may theoretically worsen metabolic imbalances and promote nephrotoxicity [10,14]. However, retrospective studies, such as the analysis by Abdel-Nabey et al., have suggested that deferring or interrupting chemotherapy in patients with TLS and coexisting renal impairment is associated with higher mortality, reinforcing the potential benefit of early tumor cytoreduction under close monitoring [14]. Indeed, urgent chemotherapy, combined with supportive measures like hemodialysis and effective uric acid control, may halt the vicious cycle of ongoing tumor turnover and further accumulation of intracellular breakdown products [10,15].

Rasburicase is regarded as the first-line agent for controlling hyperuricemia in TLS due to its rapid and efficient degradation of uric acid, yet availability, cost, and potential contraindications (e.g., glucose-6-phosphate dehydrogenase deficiency) may restrict its use [10,11,16]. In cases where rasburicase is not feasible, febuxostat has demonstrated superior efficacy to allopurinol in the prevention of TLS and reduction of serum uric acid in high-risk patients, including those with underlying renal impairment, as shown in the FLORENCE trial by Spina et al. [16]. Moreover, aggressive intravenous hydration and correction of electrolyte derangements are essential components of STLS management, along with continuous renal replacement therapy or intermittent hemodialysis in cases of refractory hyperkalemia, elevated phosphate levels, or oliguria [11,12].

Because STLS lacks specific guidelines or large prospective trials due to its low incidence, current treatment strategies are typically adapted from established recommendations for chemotherapy-induced TLS, such as those published by Coiffier et al. and Cairo et al. [11,12]. In this context, early recognition remains paramount, as STLS can rapidly progress to severe multiorgan dysfunction if diagnosis is delayed [4,8]. A focused review of five recent hematologic STLS reports (Table 3) highlights the stakes: dialysis-dependent AKI developed in 60%, and in-hospital mortality reached 80%, despite rasburicase or allopurinol use in most cases. Against this grim backdrop, our patient achieved full renal recovery and early survival after febuxostat-based urate control, continuous renal replacement therapy, and dose-adjusted R-EPOCH initiated within 72 hours of admission. This outcome demonstrates that decisive cytoreduction, coupled with metabolic support, can overturn historically poor prognoses, even when first-line rasburicase is unavailable. Nevertheless, the relative success of febuxostat in this setting exposes a critical evidence gap; hence, prospective, comparative studies are urgently needed to clarify the optimal first-line urate-lowering strategy for STLS, especially in resource-constrained centers [10,11,14].

**Table 3:** Summary of published adult cases of spontaneous tumor lysis syndrome (STLS) from hematologic malignancies AML = acute myeloid leukemia; ALK = anaplastic lymphoma kinase; BM = bone marrow; Cr = serum creatinine; CVVH = continuous veno-venous hemofiltration; CVVHD = continuous veno-venous hemodialysis; DLBCL = diffuse large B-cell lymphoma; HD = intermittent hemodialysis; NR = not reported; OD = once daily; R-EPOCH = rituximab, etoposide, prednisone, vincristine, cyclophosphamide, doxorubicin; Rx = treatment; STLS = spontaneous tumor lysis syndrome; VF = ventricular fibrillation.

Case	Age and sex	Malignancy and setting	Peak Cr (mg/dL)	Uric acid (mg/dL)	Urate-lowering Rx	Dialysis	Outcome
Our patient	56 M	Stage IV DLBCL (colon, BM, nodes)	5.2	13.4	Febuxostat 40 mg OD	CVVH × 3 days	Alive at transfer (CNS relapse later)
Riccio et al. (2006) - two AML cases [17]	62 M/74 M	De novo AML (bulky marrow)	3.4/3.1	9.9/12.6	Allopurinol	None	1 survived acute episode, 1 died day 22
Gangireddy et al. (2019) [18]	80 M	DLBCL neck mass, no chemo yet	2.74	15.9	Rasburicase (single dose)	HD day 2	Died after multi-organ failure
Tufan et al. (2005) [19]	77 M	DLBCL + Richter syndrome	9.9	16.5	Hydration ± allopurinol	Emergent HD	Died day 3 (VF/shock)
Chapman-Fredricks et al. (2014) [20]	39 M	ALK+ DLBCL, no prior therapy	4.16	9.4	Rasburicase 0.2 mg/kg × 4 days	CVVHD	Died week 6 (septic shock)

## Conclusions

Further research is necessary to refine the optimal timing and intensity of chemotherapy in STLS, particularly in patients with high tumor burdens and compromised renal function. Detailed analyses of dosing strategies for cytotoxic agents, the comparative roles of febuxostat versus rasburicase in resource-limited settings, and the thresholds for initiating renal replacement therapy will likely contribute to more standardized protocols. As knowledge about STLS grows, increased awareness among clinicians, rapid identification of high-risk patients, and aggressive management of metabolic disturbances will be crucial in limiting morbidity and mortality.
